# Ureteral Obstruction and Ureteral Jet Identification—A Case Report

**DOI:** 10.21980/J8206G

**Published:** 2021-10-15

**Authors:** Chad Bambrick, Dalia Khader, Therese Mead

**Affiliations:** * Central Michigan University College of Medicine, Department of Emergency Medicine, Saginaw, MI

## Abstract

Point-of-care ultrasound is often used as first line imaging for the evaluation of flank pain in the emergency department and can potentially expedite diagnosis or decrease the use of abdominal CT scans, preventing unnecessary radiation exposure. This is a case report of a 77-year-old female who presented with acute flank pain. Point-of-care ultrasound revealed the absence of a ureteral jet on the right side, supporting the diagnosis of ureteral obstruction, which was confirmed on computed tomography. This case report aims to demonstrate the value of identification of ureteral jets when using point-of-care ultrasound to diagnose ureteral obstruction. Knowledge of more subtle and specific ultrasound findings such as this can help expedite more precise diagnoses.

**Topics:**

Ureteral obstruction, ureteral jets, hydronephrosis, renal colic, renal calculi, point-of-care ultrasound, flank pain.

**Figure:**
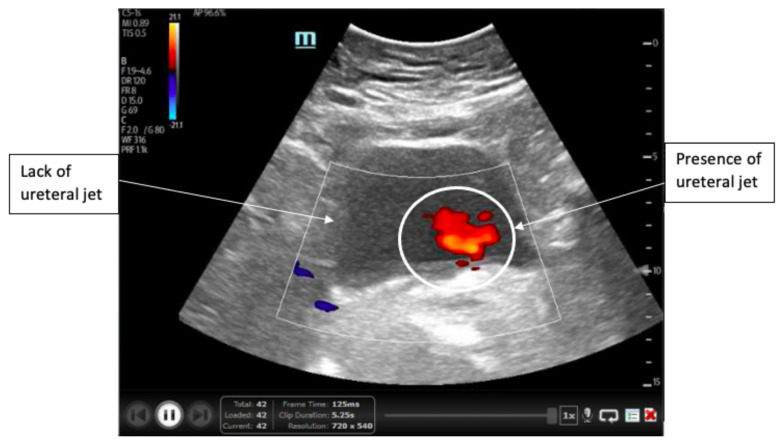
Video Link: https://youtu.be/6LeXYAP_3AQ

## Brief introduction

Renal colic is one of the most common presenting complaints in the emergency department (ED) as the prevalence of nephrolithiasis is increasing. Currently, the lifetime prevalence is 11% for men and 7% for women, affecting 1 in 11 people in the United States.^1^,^2^ Aside from predisposing biological factors such as younger age and male gender, the rising prevalence may be due to decreased fluid intake, socioeconomic status of patients affected, increased use of medications that predispose to stone formation, increased comorbidities such as diabetes and obesity, and increased use of abdominal imaging.^2^ Abdominal imaging is the standard of diagnosis for nephrolithiasis and is often used in the emergency department with complaints of renal colic. The gold standard for diagnosis of acute ureteral calculi has become abdominal computed tomography (CT), which has a sensitivity of 91–100% and specificity of 91–97%. With increased use of abdominal CT scans though, patients are becoming exposed to more radiation, increasing the risk of carcinogenesis.^3^ Additional methods of testing for ureteral calculi include plain radiography of the kidney, ureter, and bladder (KUB) or ultrasonography. KUB is more often used for follow up of stones, whereas ultrasonography is often used as a first line for imaging in the emergency department in an effort to reduce the risk of radiation exposure, costs, future ED visits or hospitalization, increased pain, and other adverse events.^4^ Ultrasound can detect the presence of perinephric fluid, hydronephrosis, or ureteral obstruction and, when ultrasonography is normal, mirrors the accuracy of CT imaging in predicting a low likelihood for urologic intervention within 90 days.^3^

Point-of-care ultrasound (POCUS) has modest diagnostic accuracy and can help detect these findings quickly in the emergency department, especially in those with moderate to severe hydronephrosis and signs of ureteral obstruction.^5^ In addition to hydronephrosis, the unilateral absence of ureteral jets may be an indication of ureteral obstruction. Inclusion of color doppler has been shown to detect the presence of ureteral jets and can be used as the first line in diagnosis and management of ureteral obstruction secondary to ureteral calculi or other obstructions. When compared to healthy individuals, there is a significant decrease in the presence of ureteral jets on the side of obstruction. In the presence of ureteral calculus with obstruction, there would be fewer or no ureteral jets noted on the side of the calculus.^6^ This case report demonstrates the use of POCUS in the diagnosis of urinary tract obstruction and the benefit of color doppler when evaluating for ureteral jets.

## Presenting concerns and clinical findings

A 77-year-old female presented to the emergency department (ED) with intermittent right-sided flank pain that started six hours prior. She described the pain as colicky in nature with a waxing and waning course. The patient reported a history of prior ureteral calculi and stated that her current pain was very similar in nature. Her only other associated symptom was nausea. She denied dysuria, hematuria, fevers or chills. Initial physical examination findings demonstrated tenderness to palpation of the right flank. The rest of the physical exam was unremarkable.

## Significant findings

A point-of-care ultrasound of the urinary tract was performed, evaluating the kidneys and bladder. When imaging her kidneys, right-sided hydronephrosis was noted with a normal appearance to the left kidney. To further evaluate, a curvilinear probe was placed on her bladder with color doppler to assess for ureteral jets. Ureteral jets are seen as a flurry of color ejecting from each of the ureters as urine is released from the ureterovesical junction. In a healthy patient, this finding should be seen ejecting from both ureters every 1–3 minutes as the kidneys continue to filter the blood and create urine to be stored in the bladder. In our patient, however, ureteral jets were only noted on the left side (arrow), which was significant in further verifying our suspicion of right ureteral obstruction.

## Patient course

Initial assessment of this patient included laboratory studies, intravenous analgesics and hydration. Given the patient’s history and physical, the diagnosis highest on initial differential was ureterolithiasis. The laboratory studies returned almost completely normal, with a normal white blood cell count and renal function tests. Interestingly, she also had a normal urinalysis with no red blood cells, which are typically seen in ureterolithiasis. This absence of hematuria, however, did not exclude the possibility of ureteral stones, which prompted further evaluation.^8^,^9^ Rather than immediate CT imaging, the decision was made to obtain a bedside ultrasound for evaluation of renal obstruction. This ultrasound imaging demonstrated mild right hydronephrosis. A curvilinear probe was then placed on the bladder with color doppler for three minutes and demonstrated the complete absence of a ureteral jet from the right ureter, despite identification of ureteral jets on the left. These findings were consistent with right-sided ureteral obstruction, which was then confirmed with a non-contrast CT of the abdomen and pelvis. These findings were further investigated and found to be secondary to right ureteropelvic junction stenosis rather than a ureteral calculus, but still demonstrated the efficacy of the absence of ureteral jet observation in the diagnosis of ureteral obstruction.

## Discussion

Point-of-care ultrasound has proven to be a valuable initial diagnostic tool in the emergency department in the evaluation of renal colic. In our patient, the specific finding of ureteral jet absence on the side of ureteral obstruction was found to be consistent with CT scan findings. Though limited, the literature investigating the use of ureteral jets as a diagnostic tool for evaluation of ureteral obstruction demonstrates a fairly high sensitivity and specificity. In a study by de Bessa et al., when defining a positive ureteral jet test as one ureter firing <25% of the total number of ureteral ejections in a 5-minute time span, the detection of ureteral obstruction had a sensitivity of 87% and a specificity of 96.4%.^7^ In another study investigating the use of doppler ultrasound detection of ureteral jets for renal colic, Pepe et al. found the sensitivity to be 94.8%.^10^ Their specificity was lower at 55.5%, likely secondary to the fact that a ureter can also be obstructed by pathology other than calculi.^10^

As in our patient, these results indicate that doppler ultrasound investigation of ureteral jets is a reliable way to quickly and effectively evaluate for ureteral obstruction with high sensitivity and specificity. When combined with CT imaging, the sensitivity and specificity of detecting ureteral obstruction from a ureteral calculus are near 100%.^10^ Ultrasonography can also aid in the reduction of radiation, particularly in younger patients, by potentially diagnosing a ureteral obstruction without the need for CT imaging. Further research likely needs to be performed before wide adoption of doppler ultrasound is implemented for this purpose, but this additional use of ultrasonography has a high potential to provide safer and quicker diagnostic evaluation and treatment of patients with ureteral obstruction.

## Supplementary Information






