# Supplementary figures and images for: Ureteral Obstruction and Ureteral Jet Identification—A Case Report
Source: J Educ Teach Emerg Med. 2021 Oct 15;6(4):V12–4. doi: 10.21980/J8206G (PMC10332735; doi:10.21980/J8206G)

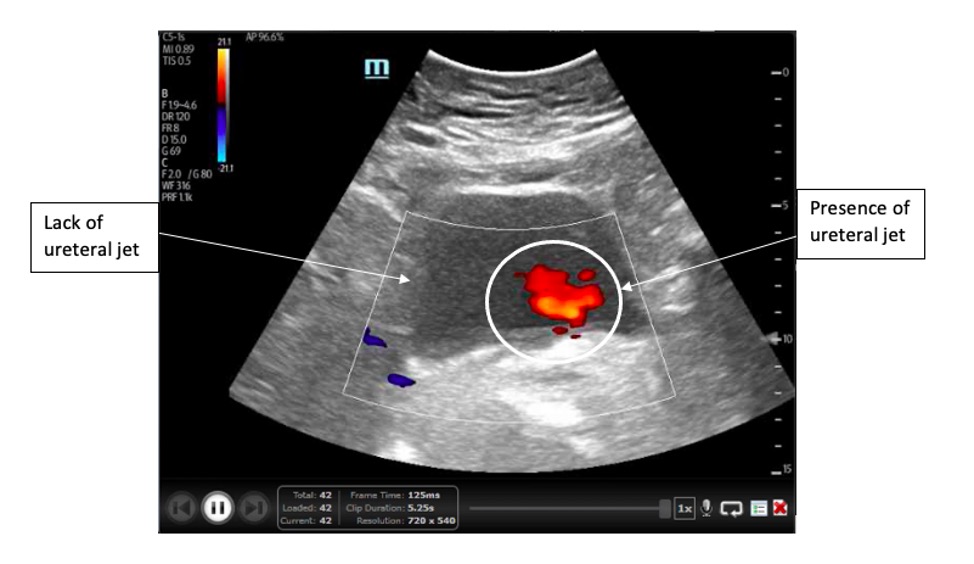

Supplement: Supplementary file 2 [file JETem-6-4-V12-supp2.jpg]

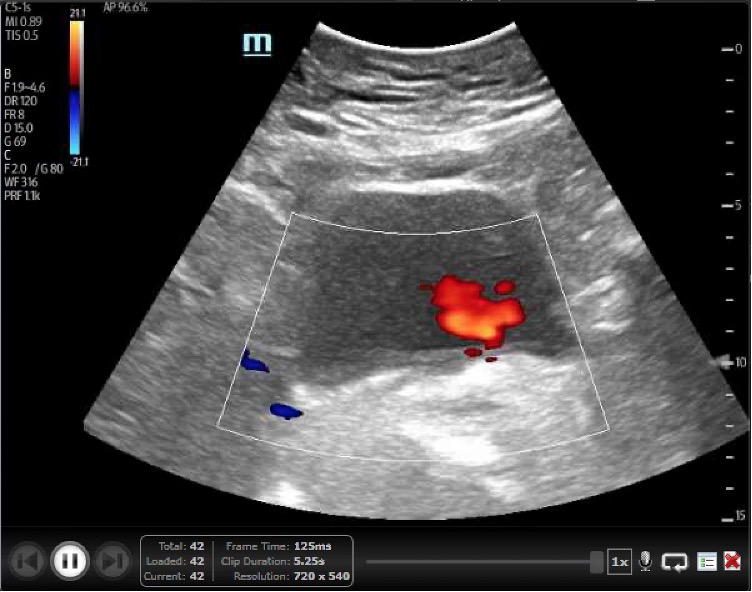

Supplement: Supplementary file 3 [file JETem-6-4-V12-supp3.jpg]
